# Molecular Interactions between Complement Factor H and Its Heparin and Heparan Sulfate Ligands

**DOI:** 10.3389/fimmu.2014.00126

**Published:** 2014-03-31

**Authors:** Stephen J. Perkins, Ka Wai Fung, Sanaullah Khan

**Affiliations:** ^1^Department of Structural and Molecular Biology, University College London, London, UK

**Keywords:** analytical ultracentrifugation, surface plasmon resonance, X-ray scattering, complement factor H, heparin, heparan sulfate

## Abstract

Complement factor H (CFH) is the major regulator of the central complement protein C3b in the alternative pathway of complement activation. A molecular view of the CFH interaction with native heparan sulfate (HS) is central for understanding the mechanism of how surface-bound CFH interacts with C3b bound to host cell surfaces. HS is composed of sulfated heparin-like S-regions that alternate with desulfated NA-regions. Solution structural studies of heparin (equivalent to the S-regions) and desulfated HS (the NA-regions) by scattering and ultracentrifugation showed that each structure was mostly extended and partially bent, but with greater bending and flexibility in the NA-regions compared to the S-regions. Their solution structures have been deposited in the Protein Data Bank. The largest HS oligosaccharides showed more bent and flexible structures than those for heparin. A folded-back domain structure for the solution structure of the 20 domains in CFH was determined likewise. CFH binds to the S-regions but less so to the NA-regions of HS. The bivalent interaction of CFH–heparin was observed by ultracentrifugation, and binding studies of CFH fragments with heparin-coated sensor chips. In common with other CFH interactions with its physiological and pathophysiological ligands, the CFH–heparin and CFH–C3b interactions have moderate micromolar dissociation constants *K*_D_, meaning that these complexes do not fully form *in vivo*. The combination of the solution structures and binding studies indicated a two-site interaction model of CFH with heparin at cell surfaces. By this, the bivalent binding of CFH to a cell surface is co-operative. Defective interactions at either of the two independent CFH–heparin sites reduce the CFH interaction with surface-bound C3b and lead to immune disorders.

## Introduction

**Figure 1:**
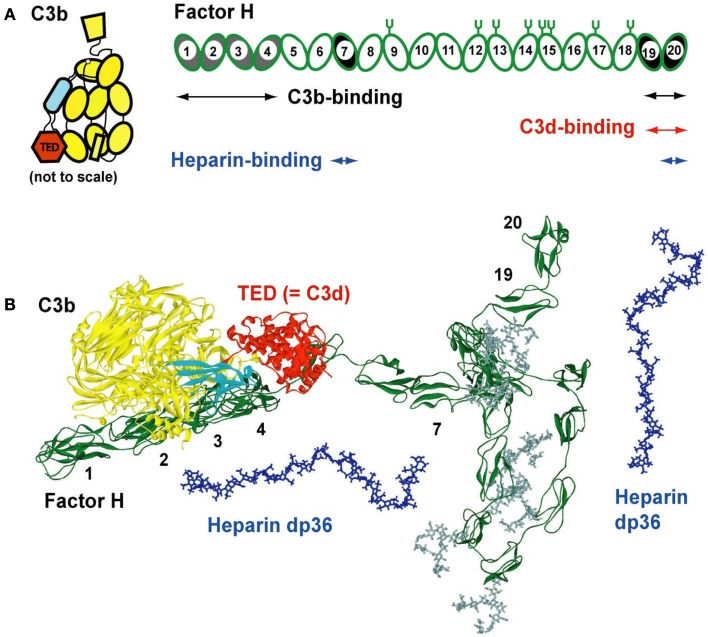
**Domain structure of factor H and its C3b and heparin ligands**. **(A)** Schematic view of the 20-SCR domains of CFH. The positions of two C3b binding sites (SCR-1/4 with C3b decay acceleration and factor I activity, and at SCR-19/20), two heparin-binding sites at SCR-7 and SCR-20, and two C3d-binding sites on each of SCR-19 and SCR-20 are shown schematically. The location of eight *N*-linked glycosylation sites is shown by symbols; a ninth site at SCR-4 is not occupied. The 12 domains of C3b are shown to the left, with the TED domain (“thioester domain”; equivalent to C3d) in red and the CUB domain (“complement C1r/CIS–UEGF–BMP1”) in cyan. **(B)** Comparison of molecular views for CFH with structures for its C3b and heparin dp36 ligands. All are on the same scale. A folded-back domain arrangement of a best-fit CFH structural model is shown with its longest length running from left to right (SCR-1–SCR-20). The CFH structure is from two recent studies (27, 54). The eight CFH oligosaccharides are shown in gray. The CUB and TED domains are shown in cyan and red, respectively. The SCR-1/4 domains are shown bound to C3b at the left (28). The folded-back central region of CFH with the shorter and glycosylated SCR domains is shown to the bottom right. Two solution structures of dp36 (blue) are shown proximate to SCR-7 and SCR-19/20.

Complement is a major defense and clearance system of the innate immune system (1, 2). Pathogens activate complement C3 through one of three pathways, the classical, lectin, or alternative pathways. Unactivated C3 consists of 13 domains, namely eight macroglobulin domains, a linker domain, an anaphylatoxin domain (C3a) a complement C1r/CIS–UEGF–BMP1 domain (CUB), a C345 domain, and a thioester domain (TED, also known as C3d). These three pathways lead to the removal of the small C3a domain from C3, the main complement protein, to convert this to C3b. Active C3b is rapidly generated through a cascade and becomes covalently attached to cell surfaces through its TED domain (Figure 1A). This triggers the assembly of the membrane attack complex that lyses pathogen cells and the clearance of C3b-opsonized cells by phagocytosis. C3b formation needs regulation, because too much C3b will damage host cells, while too little C3b means that the host becomes immuno-compromised. Recent reviews refer to this balance as a “double-edged sword” (3, 4). C3u is formed by the spontaneous hydrolysis of the thioester bridge in C3, but is no longer able to bind to cell surfaces. The main C3b regulator is complement factor H (CFH; Figure 1A), and CFH also binds to C3u. Both C3 and CFH are relatively abundant complement proteins in plasma. The most abundant plasma proteins are human serum albumin (30–50 mg/ml) and the immunoglobulins (10–15 mg/ml). In comparison, C3 is typically found at 1.0–1.6 mg/ml (5.3–8.5 μM), while CFH is almost equimolar to C3 at around 0.116–0.81 mg/ml (0.8–5.3 μM). The large amounts of these complement proteins compared to others, such as, for example, the main coagulation proteins present at 0.1–10 μg/ml (0.1–0.3 nM), are attributable to the need for abundant reagents to combat infections. The uncontrolled release of C3b is regulated by CFH. Firstly, CFH blocks the binding of complement Factor B to C3b and that of its activated form Bb to C3b, the binding of which produces the C3 convertase that cleaves C3 to produce even more C3b. Secondly, CFH acts as a cofactor of the protease Factor I that cleaves C3b into the inert fragments C3d and C3c. CFH functions both in plasma and by binding to host cell surfaces through interactions with anionic oligosaccharides bearing clusters of negative charges.

Here, we summarize recent progress in our molecular understanding of how CFH binds to heparin and heparan sulfate (HS), thereby protecting host cell surfaces. Firstly, we describe recent three-dimensional molecular structures for heparin and HS. Their original structures came from a ^1^H NMR study of heparin and the crystallography of small oligosaccharides bound to proteins. These first structures have now been supplemented by the use of a powerful new method based on the modeling of X-ray scattering curves and sedimentation coefficients that produced new structures for the larger fragments of heparin and HS. Secondly, we summarize the molecular structure for CFH. Intact CFH is comprised of 20 short complement regulator (SCR) domains, each of length about 61 residues, and joined together by linkers of lengths 3–8 residues (Figure 1A). Crystal and/or NMR structures are available for over half the CFH SCR domains. Intact CFH has not been crystallized to date, this being attributed to its comparatively large size, its ability to dimerize in at least two sites, its sizeable glycosylation in the center of CFH, and the potential flexibility of the inter-domain linkers. Instead, X-ray scattering and sedimentation coefficient modeling gave the first molecular structures for full-length CFH (Figure 1B). Thirdly, we describe the complexes formed between CFH and heparin. Again no crystal structures for CFH–heparin complexes are known to date. The application of analytical ultracentrifugation to the heparin complexes with CFH revealed the existence of bivalent CFH–heparin complexes. Binding experiments by surface plasmon resonance confirmed the existence of these bivalent complexes, and importantly showed that these complexes are formed co-operatively.

These structural advances have transformed our understanding of the molecular basis of the interactions of CFH with heparin and HS. They also provide new insight on the molecular mechanisms that lead to immune diseases such as age-related macular degeneration (AMD) and atypical hemolytic uremic syndrome (aHUS).

## Biophysical Methods

We summarize for immunologists the biophysical methods used for these studies. The joint use of three independent (or “orthogonal”) biophysical methods is a powerful means of characterizing the molecular interactions of heparin and HS with CFH. This combination reduces the experimental uncertainties inherent in the use of one method alone. For example, the dissociation constants *K*_D_ of an interaction can be obtained from all three methods as a quantitative test of consistency. In addition, each method has unique strengths.

(a) In analytical ultracentrifugation, sedimentation velocity experiments subject the samples to high rotor speeds. These, sediment to the bottom of the rotor cells at rates that depend on the macromolecular shape and mass according to the Svedberg equation. Modern PCs record as many as several hundred sedimentation boundaries during an experiment. The resulting sedimentation coefficient distribution *c*(*s*) produces peak(s) that correspond to the macromolecule(s) present in the sample (see below). When complexes are studied, the unbound and complexed species are revealed by separate peaks (if slow exchange conditions are satisfied) that provide the individual sedimentation coefficients *s*_20_,*_w_* (after correction to 20°C and the density of water) and their molecular masses. The *K*_D_ values are obtained from integration of the *c*(*s*) peak areas (5). The *s*_20_,*_w_* values report on the shape and can be compared directly with molecular models. The main advantage of this method is the ability to resolve distinct species.
(b) X-ray scattering measures the diffraction from macromolecules in random orientations in solution, from which the overall dimensions of the solution structure can be determined. Dimensions are measured using the radius of gyration *R*_G_ values from Guinier plots and the lengths from distance distribution *P*(*r*) analyses. If atomic structures are available for modeling, such as the domains of a large multidomain protein, a molecular structure can be determined by constrained modeling methods. By this, the known domain structure is rearranged into thousands of possible allowed conformations, then the best-fit molecular structure is identified by curve-fitting. This is the main advantage of scattering. The resulting structure is deposited in the Protein Data Bank as a permanent record. If scattering is used for determining the *K*_D_ value, this requires knowledge of the scattering curves for the unbound structures as well as for their complex (5). The ratio of the unbound and complexed species is determined by scattering curve fits measured at different concentrations, from which the *K*_D_ value is calculated.
(c) Surface plasmon resonance uses ligands or macromolecules that are immobilized on a sensor chip. The on-rate and off-rate of a soluble “analyte” binding to the immobilized interaction partner (the “ligand”) are determined. If these rates are relatively slow, the ratio of the off-rate/on-rate gives the *K*_D_ value. If these rates are relatively rapid, the overall intensity change of the response when the analyte is bound to the ligand as a function of its concentration given an alternative determination of the *K*_D_ value (6). No shape information is available by this method. However, a different sensor chip technology termed dual polarization interferometry (data not shown) provides both the *K*_D_ value and the dimensions of the bound analyte molecule.

## Solution Structures of Heparin dp6–dp36

**Figure 2:**
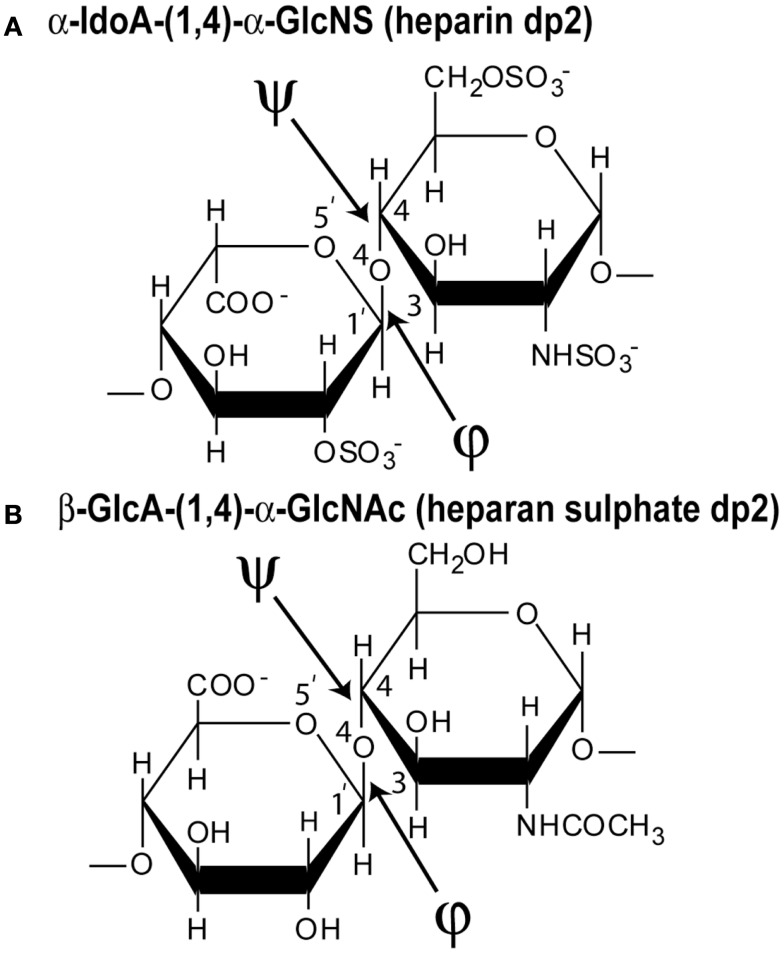
**Chemical structures of the two disaccharide repeats of heparin and HS**. **(A)** The major repeating disaccharide unit in heparin (α-iduronic acid-2-sulfate → α-glucosamine-2,6-disulfate; α-IdoA2S and α-GlcNS6S). The molecular mass of this trisulfated disaccharide is 573 Da. The φ angle between the rings is determined from the O5′–C1′–O4–C4 atoms and the ψ angle is determined from the C1′–O4–C4–C3 atoms, indicated by two arrows. **(B)** The major repeating non-sulfated disaccharide unit of HS (β-glucuronic acid → α-*N*-acetylglucosamine; β-GlcA and α-GlcNAc). The molecular mass of this averaged disaccharide is 378 Da.

The disaccharide subunit (dp2) of heparin contains two residues of uronic acid and d-glucosamine linked by a (1 → 4) glycosidic bond (Figure 2A). The uronic acid can be either α-l-iduronic acid (α-IdoA), which accounts for up to 90% of heparin, or β-d-glucuronic acid (β-GlcA), which accounts for up to 10% of heparin. A heparin disaccharide most often contains three sulfate groups, one located on the 2-OH group of α-IdoA, and two at the 2-NH_2_ group and the 6-OH group of d-glucosamine (α-GlcNS), namely [→4)-α-l-iduronic acid-(1 → 4)–α-d-glucosamine (2,6-disulfate)-(1 →]. This is abbreviated as IdoA2S–GlcNS6S. In fact, heparin is most heterogenous (7). While the main structure is the repeating tri-sulfated disaccharide IdoA2S–GlcNS6S, there are four possible uronic acids in heparin, namely GlcA, GlcA2S, IdoA, and IdoA2S, with the last one being the most common. There are seven possible glucosamines, namely GlcNS, GlcNS6S, GlcNS3S, GlcNS3S6S, GlcNAc, GlcNAc6S, and rarely glycosamine with a free amine. The most common is GlcNS6S. The other disaccharide in heparin is [→4)-β-d-glucuronic acid-(1 → 4)–α-d-*N*-acetyl glucosamine-(1 →], which is abbreviated as GlcA–GlcNAc. The high degree of sulfation and carboxylation makes heparin the most negatively charged macromolecule known in biology. For experimental studies, the six fragments of heparin dp6–dp36 were prepared from bovine lung heparin, starting from heparinase-I-digested heparin, followed by Biogel P-10 preparative gel permeation chromatography to separate the individual oligosaccharides (8).

**Figure 3:**
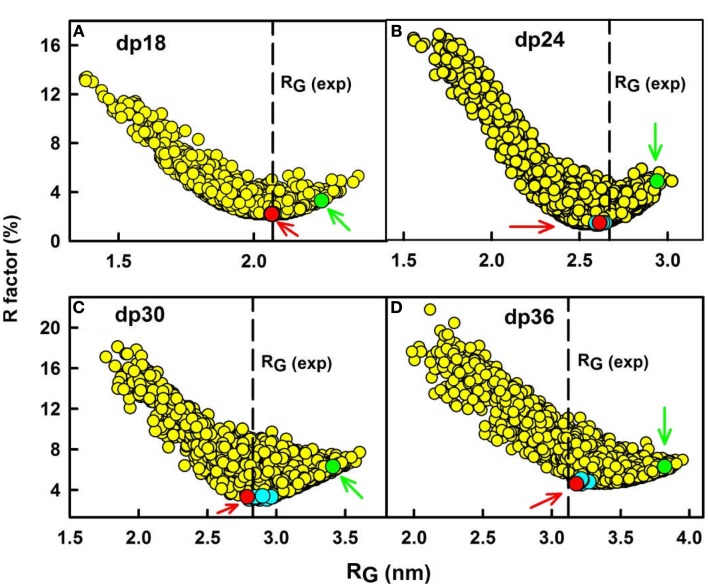
**Constrained modeling analyses of heparin dp18–dp36**. **(A)** The *R*-factor values from the curve fits for 5,000 trial randomized models of heparin dp18 are compared with their *R*_G_ values. The vertical dashed line corresponds to the observed experimental *R*_G_ value. The red circle (arrowed) denotes the best-fit model, and the green circle (arrowed) denotes the linear model for heparin. Other best-fit models are shown in cyan close to the *R*-factor minimum. Here and in Figures 4–6, the heparin structures are corrected for minor steric overlaps (9). **(B–D)** The corresponding analyses for each of the 5,000 trial models for heparin dp24, dp30, and dp36 are shown in that order.

Our ultracentrifugation and scattering fit procedure gave molecular structures for the six heparin fragments dp6–dp36. Conformational information on the structure of heparin had previously come from NMR studies of heparin dp12 and crystallography studies of proteins co-crystallized with small heparin fragments with sizes close to dp5. Our new structural analyses (8) proceeded in three stages:

(a) First, using analytical ultracentrifugation, sedimentation velocity runs were performed on each of the six heparin fragments dp6–dp36. Single clean peaks were seen in the *c*(*s*) analyses, indicating that the purifications gave satisfactorily homogenous preparations. The six experimental sedimentation coefficients increased with size, ranging from 1.09 S for dp6 to 1.84 S for dp36. Linear molecular models were created for heparin dp10–dp40 starting from the NMR molecular structure for heparin dp12. The sedimentation coefficients *s*_20_,*_w_* calculated from these linear models agreed well with the experimental values.
(b) Second, X-ray scattering curves with very good signal–noise ratios were measured at the ESRF synchrotron, even for the smallest heparin, dp6. This resulted because of the high beam intensity, low scattering backgrounds, and improved detector technology. Good linear Guinier analyses and distance distribution curves resulted in *R*_G_ values that increased from 1.03–1.33 nm for heparin dp6 to 3.12–3.52 nm for heparin dp36. The comparison with the linear heparin models showed that the modeled *R*_G_ values increased linearly with heparin size, while the experimentally measured *R*_G_ values for dp18–dp36 did not increase in proportion. Therefore, the experimental *R*_G_ values showed sensitivity to bending in the heparin structures with increase in size. Other scattering parameters (i.e., the cross-sectional *R*_G_ values and the maximum dimensions) also indicated that the larger heparin structures displayed bending.
(c) Third, constrained atomistic modeling revealed the molecular structures of heparin dp6–dp36 that best fitted the X-ray scattering curves. The major conformational determinant of heparin are the two torsion angles φ and ψ of the glycosidic linkage (Figure 2A), defined by the O5′–C1′–O4–C4 atoms and the C1′–O4–C4–C3 atoms, respectively. Both torsion angles were randomized in steps of up to ±45°. This process generated 5,000 randomized heparin structures, starting from a linear structure. Each randomized model was compared with the experimental X-ray data by calculating the *R*_G_ value of each model and the goodness-of-fit *R*-factor of each curve fit. The best-fit polysaccharide structures were determined from V-shaped graphs of *R*-factors vs. *R*_G_ values by identifying the points with the lowest *R*-factors that showed the best agreement with the experimental *R*_G_ values (red circles; Figure 3).

**Figure 4:**
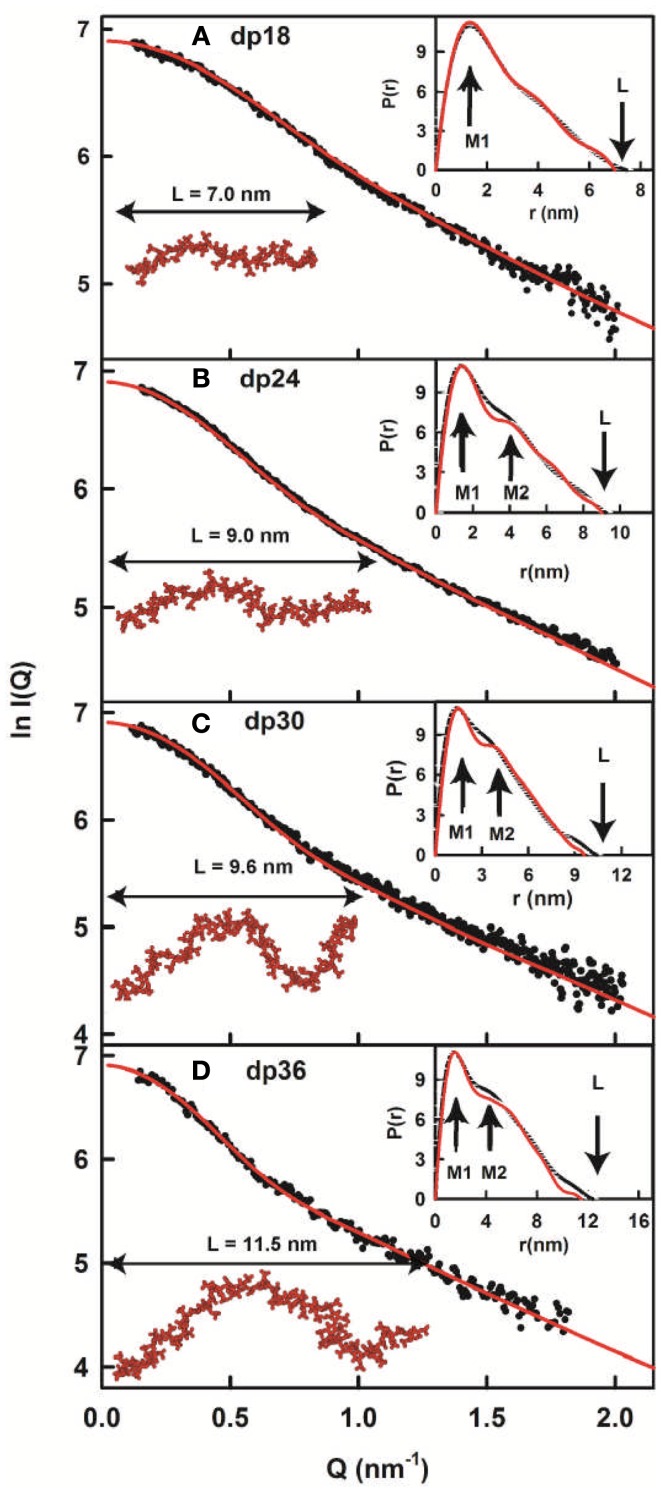
**X-ray modeling curve fits for best-fit heparin dp18–dp36 models**. **(A–D)** Correspond to the structural fits for heparin dp18, dp24, dp30 and dp36. The experimental *I*(*Q*) and *P*(*r*) X-ray scattering data are represented by black circles or lines, respectively. The red lines and models correspond to the best-fit dp18–dp36 models from the searches (Figure 3). The maximum lengths of the models are shown for comparison with their *L* values in the *P*(*r*) curves.

**Figure 5:**
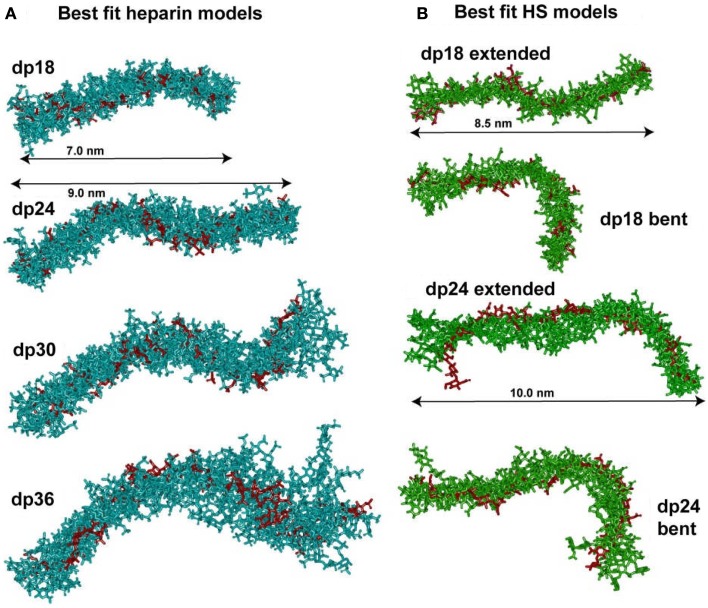
**Superimposition of the best-fit models for heparin and HS**. **(A)** The nine best-fit models for each of the four heparin fragments dp18–dp36 were superimposed. Only the non-hydrogen atoms are displayed. The best-fit model is shown in red, while the others are shown in cyan. The overall lengths of heparin dp18 and dp24 are shown as 7.0 and 9.0 nm, as arrowed. **(B)** Each set of eight best-fit models for the two HS fragments dp18 and dp24 were superimposed. The best-fit model is shown in red, while the others are shown in green. The overall lengths of the extended HS dp18 and dp24 models were shown as 8.5 and 10.0 nm, as arrowed. The extended best-fit HS structures were obtained primarily from filtering on the *R*_G_ values, while the bent best-fit structures were identified by filtering on the *R*-factor values only.

In conclusion, the best-fit molecular models for heparin showed that their bending increased with heparin size (Figure 4). This outcome extends the earliest NMR and crystallographic results for the small heparin structures that suggested that they were mostly linear. Our heparin dp6 and dp12 solution structures are mostly extended, but those for heparin dp18, dp24, dp30, and dp36 are reduced in lengths by 16–29% compared to their linear structures (Figure 5A). These bent models agree with the ultracentrifugation *s*_20_,*_w_* values although the *s*_20_,*_w_* values are not sensitive to conformational bending. The original models (8) have been updated by removing minor steric overlaps between individual atoms using a constant force field termed the DREIDING minimization (9). They are available in the Protein Data Bank at http://www.rcsb.org/pdb/ or after 2009 in PDB-formatted files in the Supplementary Material of our publications. As far as is known, the heparin study was the first successful application of atomistic scattering modeling to oligosaccharides, this method having previously been used for multidomain proteins.

**Figure 6:**
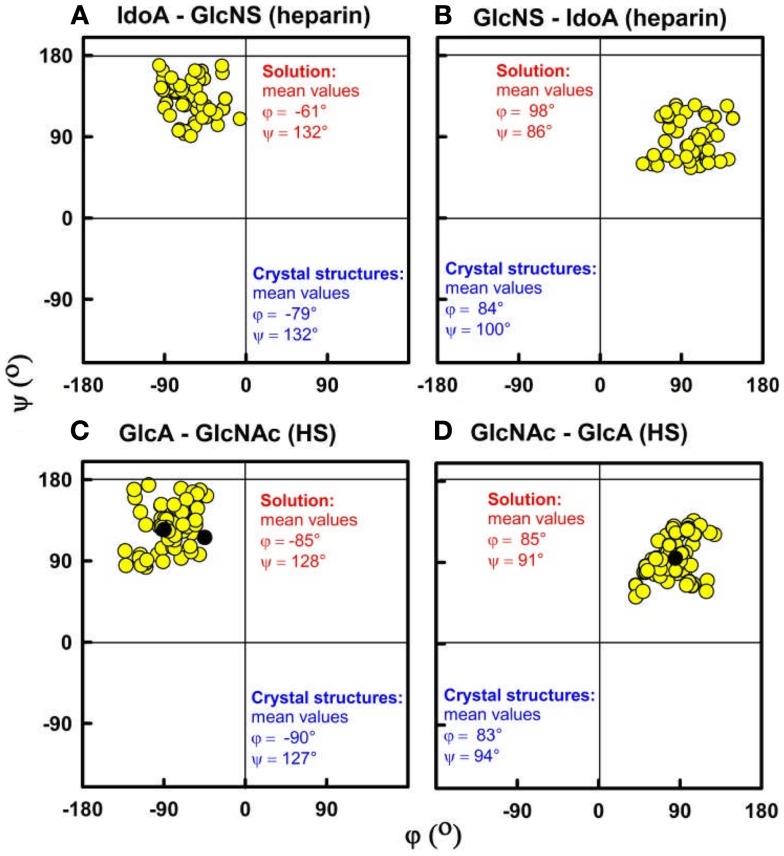
**Phi (φ) and psi (ψ) dihedral angles for heparin and HS**. The four atoms that define the φ and ψ dihedral angles are shown in Figure 2. **(A,B)** For heparin, the IdoA2S–GlcNS6S and GlcNS6S–IdoA2S φ–ψ angle pairs from the best-fit solution structure models are shown as filled circles for each of dp18, dp24, dp30, and dp36. Their mean values are shown in red (9). The φ–ψ pairs from the corresponding crystal structures are shown in blue. **(C,D)** For HS, the GlcA–GlcNAc and GlcNAc–GlcA φ–ψ angles in the best-fit models for each fragment are shown. These correspond to structures for dp6 to dp24, and their mean values are shown in red (14).

The best-fit models from varying only the φ and ψ angles between the sugar rings were readily identified at the minima in Figure 3. The quality of the dp18–dp36 scattering curve fits is better than those found with many multidomain proteins (Figure 4). From the fits, the solution φ and ψ values were around −60° and 130°, respectively, for the IdoA2S–GlcNS6S bond, and around 100° and 85°, respectively, for the GlcNS6S–IdoA2S bond (Figures 6A,B). Interestingly, comparisons with 19 crystal structures of heparin dp4–dp10 in protein complexes revealed similar φ and ψ angles (Figures 6A,B). When the outliers were removed, the crystallographic φ and ψ values were −79° and 132°, respectively, for the IdoA2S–GlcNS6S bond, and 84° and 100°, respectively, for the GlcNS6S–IdoA2S bond (Figures 6A,B). Given standard deviations of typically ±20°, these φ and ψ values suggest that the free heparin solution structures for dp18–dp36 is essentially unchanged in conformation from small heparin fragments bound to proteins in crystal structures. Thus, heparin in both its complexes or free in solution has a semi-rigid and extended conformation that is optimal for binding to proteins without major conformational changes.

The repulsion of the sulfate and carboxylate groups in heparin may influence these extended structures. The anionic sulfate and carboxylate groups of heparin form mostly ionic contacts with basic amino acids on protein surfaces. The axial orientations of these groups in heparin are thus crucial, and these repeat themselves after every four oligosaccharide rings. The dp4 fragment possesses at least six sulfate groups and two carboxylate groups. In dp4, two sulfate groups are located in opposed axial orientations in GlcNS6S, while the third sulfate and the carboxylate group are located in opposed axial orientations in IdoA2S residues (8). When crystal structures of dp3 fragments are viewed centered on either IdoA2S or GlcNS6S, these axial orientations are largely preserved and aligned with each other between IdoA2S and GlcNS6S residues (8). Our solution structures of heparin dp18–dp36 suggest that these extended arrangements and orientations seen in crystal structures are mostly preserved.

The larger heparin fragments show more pronounced bending, even though the mean φ and ψ angles are unaffected. The scattering modeling represented this bending as occasional kinks in the heparin structure. These bends may arise from the occasional occurrence of GlcA–GlcNAc residues in the dominant IdoA2S–GlcNS6S structure. It is more likely that kinks occur naturally within the IdoA2S–GlcNS6S structures, these having been observed in several protein–heparin crystal structures (8). Thus the observation of bending indicates that heparin is not completely rigid, and that limited flexibility in heparin is permitted.

The heparin structures clarify three different scenarios for heparin–protein interactions. As far as is known, heparin binding sites are generally found at surface-exposed positions in proteins. One scenario involves two independent and different heparin binding sites on the same protein, such as that in CFH. Because heparin is semi-rigid and extended, it is unlikely that a single large heparin molecule can undergo large conformational changes in order to bind both CFH–heparin sites simultaneously to form a 1:1 complex, thus these two sites remain independent (10). In a second scenario, heparin will mediate conformational changes in proteins in order to induce functional activity. The best-known case is that of antithrombin, in which the allosteric activation of its β-sheet structure by heparin lead to the inhibition and regulation of the blood coagulation protein Factor IXa, Factor Xa, and thrombin (11). A third scenario is the ability of heparin to enable functionally active protein dimers to form through heparin binding to single sites on each protein monomer [for example, the fibroblast growth factor family and their receptors (12, 13)]. All three scenarios are facilitated by the semi-rigid and extended structures of heparin.

## Solution Structures of Heparan Sulfate dp6–dp24

Native HS plays key roles in the regulation of physiological and pathophysiological processes. Native HS is comprised of the same two alternating residues of uronic acid and d-glucosamine residues as in heparin, but with a reduced degree of sulfation (Figure 2B). HS contains a higher proportion of the GlcA–GlcNAc disaccharide compared to the sulfated IdoA2S–GlcNS6S disaccharide in heparin considered above.

The overall structure of native HS shows a distinct three-domain organization that is comprised of short S-domains with IdoA2S–GlcNS6S disaccharides, long NA-domains with GlcA–GlcNAc disaccharides, and mixed domain regions at the junctions between the S-domains and NA-domains that includes IdoA2S–GlcNS6S disaccharides. The S-domains and mixed domain regions occur as “hypervariable” regions that lead to different HS functions when HS is purified from different cell types. The S-domains correspond closely to heparin. For experimental studies of the NA-domains, the non-sulfated HS oligosaccharide fragments dp6–dp24 were prepared by digests, and this product is termed the HS fragments below, unless specified otherwise. First, exhaustive heparinase I digestion was used to minimize the content of fully sulfated heparin sequences in a crude glycosaminoglycan mixture. This generated the NA-regions dp6-dp24 that remained following the removal of sulphated oligosaccharides, then Biogel P-10 preparative gel permeation chromatography was performed to separate these individual GlcA–GlcNAc oligosaccharides. The HS fragments dp6–dp24 were submitted to the same ultracentrifugation–scattering-modeling strategy used above for heparin.

Analytical ultracentrifugation on each of the HS fragments dp6–dp24 revealed homogenous preparations from the single peaks seen in the *c*(*s*) analyses. The experimental sedimentation coefficients *s*_20_,*_w_* increased with HS size from 0.82–1.05 S for dp6 to 1.26–1.35 S for dp24 as expected. This time, their values were generally reduced compared to the *s*_20_,*_w_* values for heparin dp6–dp36. Most of this reduction comes from the lower masses of HS dp6–dp24 compared to heparin dp6–dp24 (Figure 2). The remaining reduction is attributable to a more compact solution structure for the HS fragments compared to heparin, i.e., HS is not as extended as heparin. Linear molecular models were created for HS dp6–dp30 starting from a HS dp4 crystal structure. Again the experimental *s*_20_,*_w_* values were lower than the predicted values from the linear HS models, showing that HS does not have an extended structure (14).

X-ray scattering curves for the HS fragments dp6–dp24 gave *R*_G_ values that increased from 0.98–1.03 nm for HS dp6 to 2.82–3.00 nm for HS dp24. The comparison with the linear HS models showed that the predicted *R*_G_ values increased linearly with HS size, while the experimental *R*_G_ values starting from dp18 did not increase by as much. Thus the experimental *R*_G_ values also revealed bending in the larger HS structures. Bending was also indicated from the cross-sectional *R*_G_ values and the distance distribution curves *P*(*r*) (9, 14).

The constrained scattering modeling identified molecular structures for the eight HS dp6–dp24 fragments. Totals of 5,000–12,000 conformationally randomized structures were generated by variations of the two torsion angles φ and ψ. Each model was compared one-by-one against the X-ray curve. For HS dp6–dp16, good curve fits were obtained with almost linear structures with slight bending (cf: Figure 4; not shown). Interestingly, the two largest HS fragments dp18 and dp24 showed a different outcome. The *R*-factor vs. *R*_G_ graphs (cf: Figure 3; not shown) for dp18 and dp24 showed that the modeled *R*_G_ value at the minimum *R*-factor was different from the experimental *R*_G_ value. The smaller modeled *R*_G_ value of 2.13 nm (dp18) and 2.47 nm (dp24) suggested that the HS structures were noticeably bent, while the larger experimental *R*_G_ value of 2.34 nm (dp18) and 2.82 nm (dp24) suggested that the HS structures were mostly extended. The modeled *s*_20_,*_w_* values for both the bent and extended structures were the same within error. This outcome suggested that conformational heterogeneity was present in HS, and that each of HS dp18 and dp24 exhibited both bent and extended structures simultaneously in solution (Figure 5B).

The organization of sulfated heparin-like S-domains and unsulfated NA-domains in the HS structure has been clarified by our work. The comparison of our heparin and HS structures becomes essentially that between S-domains and NA-domains (Figure 5). The greater bending and flexibility of HS compared to heparin is attributable to the GlcA–GlcNAc disaccharides in HS and the IdoA2S–GlcNS6S disaccharides in heparin. The available crystal structures show that the separations between the rings in GlcA–GlcNAc and IdoA2S–GlcNS6S are similar. Thus the different heparin and HS conformations must result from altered φ and ψ angles. The φ and ψ angles for heparin dp18–dp36 in solution are within 18° of those in 19 crystal structures (Figures 6A,B). The φ and ψ angles for HS dp6–dp24 in solution were all within 5° of those in the HS dp4 crystal structure (Figures 6C,D). While not significantly larger than the standard deviations, the largest differences between heparin and HS involve the two φ angles rather than the two ψ angles. The physical basis for this is most likely to arise from the IdoA2S–GlcNS6S sequences in heparin compared to the GlcA–GlcNAc sequences in HS. Unlike HS, heparin will be influenced by greater repulsions between pairs of sulfate–sulfate, sulfate–carboxylate, and carboxylate–carboxylate groups. Thus, the combination of the NA- and S-domains within native HS suggests that different native HS structures with greater or lesser bending may arise through variations of the sizes of the NA-domains and S-domains. For example, a three-part native HS structure comprised of a NA-region at its center and flanked by two S-regions would show conformational flexibility in its central region and influence its immune reactivity.

An important caveat in these molecular studies should be noted. When we first published our HS best-fit structures (15), the anomeric configurations of GlcA and GlcNAc in our HS structures should have alternated between α and β. Unfortunately, the anomeric configurations were all β in our original HS models, thus our original study was withdrawn. The HS structures were remodeled with the correct anomers and republished (14). Although we take full responsibility for our mistake, the error was traced back to a misunderstanding of the starting HS dp4 structure in the Protein Data Bank. That HS dp4 structure was written out with its reducing end at the left, when it is more conventional to write this at the right end. In addition, terminological inconsistencies exist in the Protein Data Bank that contributed to our misunderstanding. For example, the α-GlcNAc and β-GlcA anomers of HS should have been written as NDG and BDP, respectively, in the HS dp4 crystal structure, and not as NAG and GCU (PDB code 3E7J; dated 18 August 2008). Related discrepancies in carbohydrate structures have been reported by others (16, 17). It appears essential to check carbohydrate structures from the Protein Data Bank before using these in molecular modeling.

## Ligand Interactions of Complement Factor H

We first summarize the immune functions of CFH. The 20-SCR domains of CFH perform several functions by binding to various ligands. The major N-terminal and C-terminal binding sites for complement C3b in CFH are located at SCR-1/4 and SCR-19/20, and the C3d fragment of C3b binds to SCR-19/20 (Figure 1A) (18). A third C3b binding site specific for C3c has been proposed in the central part of CFH (18–20). These interactions lead to the regulatory breakdown of C3b by CFH. The two heparin binding sites of CFH are located close to SCR-6/8 and SCR-19/20 (18). These sites enable the binding of CFH to host cell surfaces, but not to the surfaces of pathogens, thus leading to the complement regulatory protection of host cells. CFH also binds C-reactive protein at SCR-6/8 and SCR-16/20 (6). The binding of C-reactive protein to damaged host cells will lead to C3b regulation following CFH binding to C-reactive protein. Weak zinc binding sites are primarily located within SCR-6/8 (21); these sites lead to the precipitation of CFH–C3b complexes in the pathophysiological concentrations of zinc found in the retina (22). In addition to these ligand interactions, CFH self-associates with itself to form dimers and higher oligomers (23). Although the physiological role of CFH oligomers is not yet clear, except perhaps in facilitating the development of drusen deposits in the retina at the onset of AMD (23), CFH oligomer formation is a significant factor in the design of experiments with CFH.

For immune function, the four most notable features of CFH–ligand interactions are their moderate binding strengths, their multivalency, their dependence on ionic strength, and CFH self-association:

(i) The strength of the CFH–ligand interaction is central for complement regulation. For most macromolecular interactions, the dissociation constant *K*_D_ is similar to the physiological concentration. The *K*_D_ corresponds to the concentration at which a given complex is 50% dissociated. Given that C3 and CFH occur at 2–7 μM levels in plasma, the first compilation of all the CFH–ligand *K*_D_ values (24) unsurprisingly showed that most *K*_D_ values are also micromolar. These micromolar values mean that only partial complexes of CFH with C3b and heparin are formed during normal CFH regulatory function. In addition, these micromolar affinities mean that these CFH–ligand interactions can be easily misinterpreted in biochemical assays for reason of incomplete binding.
(ii) Complement factor H undergoes multivalent interactions with its major C3b, C3d, heparin, and CRP ligands. Multivalency means that analyses based on simple 1:1 interactions may not be adequate for accurate quantitative studies of the CFH–ligand interaction. This issue is best resolved by performing CFH fragments–ligand studies alongside studies based on full-length CFH, such as those of CFH with heparin to characterize its co-operative binding (10).
(iii) Many CFH–ligand interactions involve opposing ionic interactions between CFH and its ligands. The buffer in plasma corresponds to 137 mM NaCl/11 mM phosphate (24). Experiments that use low salt will promote these interactions, while high salt will inhibit these interactions. While the use of low (50 mM NaCl) and high (250 mM NaCl) salt levels can be useful, they may lead to undesired side-effects. For example, C3d is monomeric in 137 mM NaCl, yet forms dimers and trimer/tetramer in 50 mM NaCl buffer (25). A related case is 2 mM calcium that stabilizes C-reactive protein, thus the use of 2 mM calcium is important for its binding studies with CFH (6).
(iv) Full-length CFH is well-characterized to undergo 5–14% self-association in 137 mM NaCl/11 mM phosphate buffer. The presence of CFH oligomers will complicate experiments that require high protein concentrations.

## Bivalent and Co-Operative Binding of CFH–HS

The immune function of CFH is determined by its major functional activities at its N-terminal and C-terminal SCR domains (Figure 1). The middle SCR domains possess shorter sequences, longer inter-domain links, and higher glycosylation levels, suggesting that these middle domains act as conformational spacers. Because there is no crystal structure for full-length CFH, the combination of X-ray and neutron scattering and sedimentation coefficient modeling produced the first molecular structures for full-length CFH (26, 27). The CFH structure determination approach is similar to those for heparin and HS fragments above. The scattering modeling was based on molecular structures for all 20-SCR domains. These were taken directly from crystal or NMR structures for small CFH fragments, or from predicted SCR structures by homology modeling based on the closest match with known crystal or NMR structures. The inter-SCR linkers are variable and not predictable in their conformation. For the scattering modeling, these linkers were conformationally randomized and used to assemble 2,000 SCR models for CFH in randomized orientations. The scattering curve fits showed that only folded-back domain structures with an overall length of 40 nm (Figure 1B) fitted the CFH scattering data. Its length is much reduced compared to a hypothetical linear structure for CFH, which would be 73 nm in length. Two distinct models for CFH have been computed, which give similar and indistinguishable scattering fits. In the first one, the SCR-1/7 domains are extended in shape and SCR-8/20 are looped back (Figure 1) (24). In the second one, the SCR-13/20 domains are extended and SCR-1/12 are looped back (27). The first model is more easily docked with the C3b–CFH crystal structure, because this requires SCR-1/4 to be extended in shape (28).

The CFH–HS interaction forms the basis for the protection of the native HS-coated host cell surfaces via the S-domains of HS from attack by the innate immune system, directing this immune response instead against pathogenic bacteria, which lack a polyanionic oligosaccharide coating and are therefore unprotected by CFH. Polyanionic molecules such as HS and others such as the sialic acids on host cell surfaces enhance the regulatory effectiveness of CFH by 10-fold through its inhibition of complement activation (29–31). Two independent heparin binding sites are located at the SCR-6/8 and SCR-19/20 domains in CFH (Figure 1A). Notably, SCR-7 and SCR-20 have the two most basic charge densities in CFH (27), these basic charges being optimal for interactions with anionic S-domains. The availability of molecular structures for CFH, heparin, and HS fragments (above), and the combined application of ultracentrifugation, scattering, molecular modeling, and surface plasmon resonance (below) to study CFH–heparin complexes provided the first molecular picture of CFH binding to heparin.

**Figure 7:**
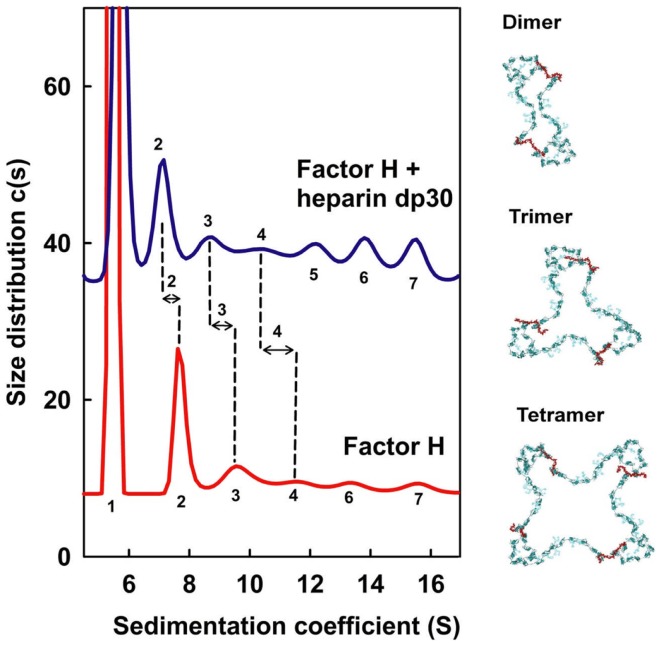
**Sedimentation velocity analyses of CFH mixtures with heparin dp30**. CFH was studied alone (red) and with a 1:1 molar ratio of dp30 (blue) by sedimentation velocity in the analytical ultracentrifuge. The CFH monomer *s*_20_,*_w_* peak is labeled as 1, and the CFH oligomer *s*_20_,*_w_* peaks for the dimer, trimer, and tetramer onward are denoted by 2, 3, 4, and upwards. Peaks 2, 3, and 4 are better defined and the change in the sedimentation coefficients between CFH and CFH–dp30 is shown by the vertical dashed lines. On the right, dimer, trimer, and tetramer models for CFH are shown in blue–green, with the heparin cross-links shown in red. The dp30 heparin sizes are sufficiently long enough to cross-link the SCR-7 and SCR-20 domains between different CFH molecules, leading to the formation of dimer, trimer, and tetramer as ring-like molecular structures. Redrawn from Khan et al. (10).

The ultracentrifugation experiments of CFH mixtures with heparin dp6–dp36 (equivalent to S-domains) identified multiple different CFH–heparin complexes in the size distribution analyses *c*(*s*) (10). Unbound monomeric CFH shows a peak close to 5.5 S. The peaks for heparin dp6–dp36 are between 1.1 and 1.8 S and do not overlap with that for CFH. Even though unbound CFH showed small oligomer peaks between 8 and 16 S that make up 15% of the total peak intensity, the marked differences in these peaks after adding heparin provided unequivocal evidence of complex formation (Figure 7). The CFH mixtures with dp6 and dp12 showed small decreases in these peaks between 8 and 16 S (not shown). However the CFH mixtures with dp18–dp36 showed large peak intensity increases of up to 63% for dp36. If CFH and heparin dp30/dp36 formed a 1:1 complex, the increased peak sizes corresponded to a *K*_D_ value of about 0.5 μM. Even with 63% CFH–heparin oligomer formation, the CFH monomer peak continued to be visible, showing that complex formation is incomplete. Ultracentrifugation also showed that heparin dp10 bound tightly to SCR-6/8 and this is consistent with a micromolar affinity (32). In addition, the small CFH oligomer peaks shifted to lower *S* values after adding heparin (Figure 7). This showed that a new type of CFH oligomer structure with more extended structures had formed with heparin. As controls, experiments with native HS material (containing both S-domains and NA-domains) showed similar peak changes, while the use of NA-domains alone showed much reduced interactions with CFH.

The scattering experiments were complementary to ultracentrifugation because of their sensitivity to aggregate formation that may be missed in the ultracentrifuge. In agreement with ultracentrifugation, little changes were seen by scattering for CFH mixtures with heparin dp6 and dp12 (10). However, large increases in both molecular weights and *R*_G_ values were seen for CFH mixtures with heparin dp18–dp36. The distance distribution analyses *P*(*r*) showed that the maximum dimension of CFH increased from 34 to 40 nm in the presence of heparin dp36. Because no indefinitely sized aggregates were seen by scattering, these results agree with the formation of large CFH–heparin oligomers with specific sizes.

**Figure 8:**
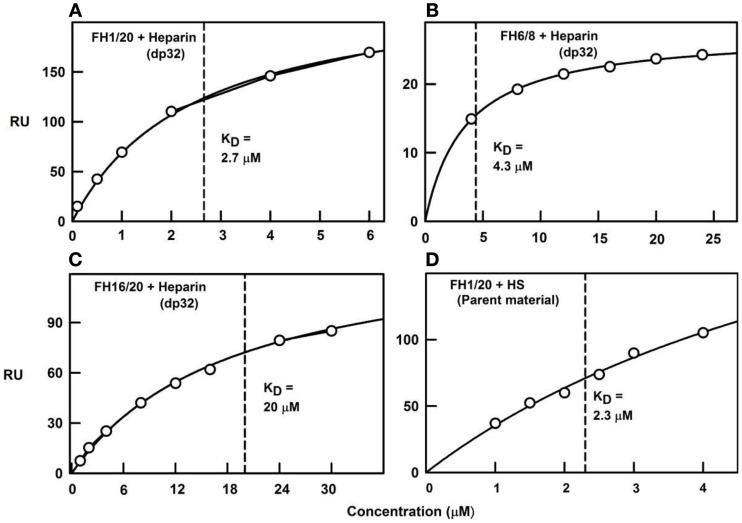
**Surface plasmon resonance of the CFH interaction with heparin and HS**. The binding curve fits used to determine the *K*_D_ values are shown for four experiments using immobilized heparin over which the CFH samples were flowed. Redrawn from Khan et al. (10). **(A)** Biotinylated heparin dp32 was immobilized on a streptavidin chip, and studied using plasma CFH to give a *K*_D_ value of 2.7 μM. **(B)** CFH SCR-16/20 was used with the same dp32 chip, from which the *K*_D_ value was determined as 20 μM. **(C)** CFH SCR-6/8 was used with the same dp32 chip, from which the *K*_D_ value was determined as 4.3 μM. **(D)** Biotinylated unfractionated HS was immobilized on a streptavidin chip, and studied using plasma CFH to give a *K*_D_ value of 2.3 μM.

To clarify the ultracentrifugation and scattering results, surface plasmon resonance experiments were performed with both full-length CFH and recombinant SCR-6/8 and SCR-16/20 in solution with heparin immobilized on the sensor chip. The binding data showed mostly fast on-rates and off-rates with the heparin surface. Curve fits of the binding responses gave *K*_D_ values of 1–3 μM for full-length CFH, and similar but significantly weaker *K*_D_ values of 4 μM for the SCR-6/8 fragment and 20 μM for the SCR-16/20 fragment (Figure 8). These *K*_D_ values confirm cooperativity between the two different heparin binding sites in CFH. As controls, the use of immobilized unfractionated HS containing both NA-domains and S-domains showed a *K*_D_ value of 2 μM with full-length CFH, while use of immobilized HS fragments (i.e., NA-domains) showed much weaker CFH binding. These experiments confirmed the importance of the heparin-like S-domains for this interaction.

These methods show that the immune function of CFH at host cell surfaces is well-described by a bivalent and co-operative model of CFH binding to the S-domains of HS. To visualize this model, molecular modeling of the ultracentrifugation *s*_20_,*_w_* data was performed. In the folded-back CFH solution scattering model (Figure 1B), the heparin binding SCR-7 and SCR-20 domains were separated by 26 nm. In contrast, the best-fit slightly bent heparin dp36 model is only 12 nm long. Thus, heparin dp36 is not long enough to cross-link the two heparin binding SCR domains in a single CFH molecule. If however the SCR-7 and SCR-20 domains in different CFH molecules were daisy-chained in alternation with heparin dp36, ring-like models for dimers, trimers, tetramers, and pentamers of CFH with heparin could be created (Figure 7). These CFH–heparin models explained the experimental *s*_20_,*_w_* values for the oligomer peaks (10) within an acceptable error of ±0.2 S (blue; Figure 7). Either of the two distinct models for CFH (see above) may be used to create these ring-like models. The modeling therefore explained the changes in the peak positions after adding heparin dp36. The modeling also explained why dp6 and dp12 did not form oligomers with CFH, i.e., because they were too short to cross-link CFH.

Complement factor H flexibility at its central domains is unlikely to play a role in heparin binding. Our experimental data for the heparin/HS fragments and CFH indicate that neither possesses sufficient flexibility to form one-to-one complexes. No increased *s*_20_,*_w_* value for the CFH monomer was seen that would indicate that CFH underwent a significant compaction in the presence of heparin (apart from relatively low changes caused by the increased mass of the CFH–heparin complex). This lack of CFH flexibility concurs with the little conformational variation seen in CFH with change in salt or pH (27). It is likely that the heavily glycosylated and smaller SCR-12/15 domains at the center of CFH maintain the structural independence of its N-terminal and C-terminal ends. The presence of these two different heparin sites at opposite sides of the CFH central region enables CFH to bind selectively, bivalently, and co-operatively to host cells showing a sufficient density of polyanions at their surfaces.

Flexibility in native HS is defined by the presence of semi-rigid S-domains and flexible NA-domains that were deduced from the scattering analyses. Our binding studies show that CFH binds to heparin-like S-domains, but less so to the non-sulfated NA-domains. If the parent HS structure shows flexibility at the NA-domains, this would enable the individual S-domains to reorientate themselves to optimize their stronger contacts with CFH. Thus CFH interacts in the same way with native HS as many other proteins do that bind to native HS through the S-domains (33).

**Figure 9:**
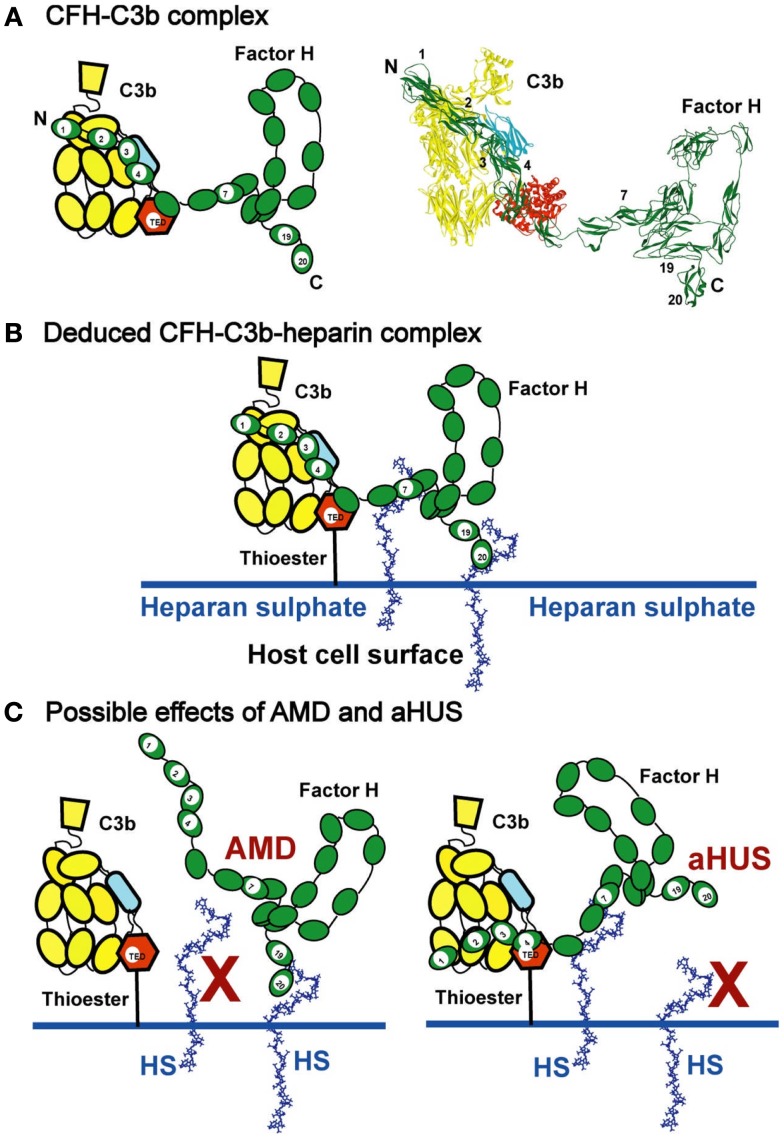
**Schematic views of CFH binding to a host cell surface**. **(A)** CFH is represented by a cartoon showing the 20-SCR domains, and bound to C3b in the cleft formed between the CUB/TED domains (blue–red) and the macroglobulin, C345, and linker domains (yellow). A molecular view of this interaction is shown on the right. **(B)** CFH binds bivalently at its SCR-7 and SCR-20 domains to anionic charges on the HS-coated host cell surface (denoted as HS and represented here by heparin dp36). For simplicity, the C-terminal interaction of CFH with C3d (Figure 1A) is not shown. This bivalent binding positions the SCR-1/4 domains in a conformation that binds readily to C3b when C3b is covalently bound to host cell surfaces through the thioester bond. The C3b interaction with CFH results in the Factor I-mediated degradation of C3b to form C3d (the TED domain) and C3c, thereby regulating complement activation at host cell surfaces. **(C)** The potential effect of polymorphisms and mutations on C3b regulation is schematically indicated. In AMD, the association of the Tyr402His polymorphism in SCR-7 is presumed to weaken the interaction between HS and SCR-7, so the proximity of CFH in relation to SCR-1/4 of surface-bound C3b is reduced. In aHUS, mutations in SCR-19 and SCR-20 are presumed to weaken the interaction between HS and SCR-19/20, while CFH remains bound to HS through SCR-7. In both AMD and aHUS, the effect is that CFH is no longer held in an optimal position on the host cell surface to interact with C3b at the SCR-1/4 domains.

The quantification of the CFH–HS interaction permits critical comparisons with other CFH–ligand affinities. The first full set of *K*_D_ values for CFH–ligand complexes was compiled recently (24). This summary (Table 1) currently shows that the strongest CFH interactions involve heparin and C3b/C3u. The affinity of CFH (concentrations of 2–5 μM in plasma) for heparin is around 1–2 μM, with the CFH binding for heparin being bivalent. These micromolar *K*_D_ values mean that the CFH–heparin complexes will not be fully formed in plasma, and the free and bound forms will be in equilibrium with each other (footnote, Table 1). The larger *K*_D_ values of 4 and 20 μM for the separate SCR-6/8 and SCR-16/20 fragments correspond to weaker binding to heparin at each of two independent binding sites (10). In comparison with the *K*_D_ value for intact CFH, this shows co-operative binding. The CFH interactions with C3b and C3u have low *K*_D_ values of around 1 μM. Since C3 is 5 μM in plasma, these complexes (Figure 9A) will again be only partially formed in plasma. The *K*_D_ values of around 10 and around 4 μM for the separate SCR-1/4 and SCR-19/20 sites for C3b, respectively, are also weaker (18). The reason why the latter two *K*_D_ values are weaker compared to intact CFH is not clear, because it has not been shown experimentally that CFH binds bivalently to one molecule of C3b or C3u. A CFH–C3b complex formed through SCR-1/4 binding (Figure 1B) may also bind to a second molecule of C3b at its C3d (TED) domain, hence decreasing the observed *K*_D_ value (Figure 1A). Overall, however, the similar binding affinities of CFH for C3b/C3u and heparin indicate that these values are optimal for the effective immune role of CFH as a host cell surface regulator of C3b activity.

**Table 1:** **Selected dissociation constants, *K*_D_, for the CFH interactions with its ligands**.

Interaction	*K*_D_ (μM)^a^	Method^b^	Buffer (abbreviated)^c^	Reference
**CFH–HEPARIN**				
CFH–heparin dp32/dp36	0.5, 2.7	SV, SPR	10 mM HEPES with 137 mM NaCl, pH 7.4	(10)
SCR-6/8–heparin dp32	4.3	SPR	10 mM HEPES with 137 mM NaCl, pH 7.4	(10)
SCR-16/20–heparin dp32	20	SPR	10 mM HEPES with 137 mM NaCl, pH 7.4	(10)
**CFH–C3b OR CFH–C3u**				
CFH–C3b	0.59–1.6	SPR	10 mM HEPES with 150 mM NaCl, pH 7.4	(18)
CFH–C3u	0.59	SV	PBS with 137 mM NaCl, pH 7.4	(34)
SCR-1/4–C3b	11	SPR	PBS with 150 mM NaCl, pH 7.4	(28)
SCR-1/4–C3b	9.8–13.5	SPR	10 mM HEPES with 150 mM NaCl, pH 7.4	(18)
SCR-19/20–C3b	5.4	SPR	PBS with 140 mM NaCl, pH 7.3	(20)
SCR-19/20–C3b	3.5–4.5	SPR	10 mM HEPES with 150 mM NaCl, pH 7.4	(18)
SCR-19/20–C3b	0.54	SPR	10 mM HEPES with 150 mM NaCl, pH 7.2	(35)
**CFH–CRP**				
CFH–CRP	4.2	SPR	10 mM Tris, 137 mM NaCl, 2 mM CaCl_2_, pH 7.4	(6)
**CFH SELF-ASSOCIATION**				
CFH–CFH	28	SE	10 mM HEPES with 137 mM NaCl and EDTA, pH 7.4	(23)
**CFH–ZINC**				
CFH–zinc	~10	SAXS	10 mM HEPES, 137 mM NaCl, pH 7.4	(21, 23)
C3b–zinc	~100	SAXS	10 mM HEPES, 137 mM NaCl, pH 7.4	(22)

*^a^ If the interacting species are both at 5 μM and the *K*_D_ value is 1 μM, 64% of the complex will be formed. If the *K*_D_ value is 10-fold weaker at 10 μM, 27% of the complex will be formed*.

*^b^ ED, equilibrium dialysis; SAXS, small-angle X-ray scattering; SE, sedimentation equilibrium; SPR, surface plasmon resonance; SV, sedimentation velocity*.

*^c^ Buffer additives are common, especially for SPR studies. These are reported in the more detailed survey of Perkins et al. (24)*.

The other CFH–ligand interactions are weaker than those of CFH–heparin. C3d (*K*_D_ of 3–8 μM) and C-reactive protein (*K*_D_ of 4 μM) (6, 36) show weaker affinities for CFH (Table 1), suggesting that these interactions only become important during excess inflammation (acute phase response) when high local concentrations of C3d or C-reactive protein may occur. When either of these two ligands is bound to host cell surfaces, additional CFH binding sites are potentially available to reinforce host cell protection, similar to that suggested for CFH–heparin (Figure 9B). The monomer–dimer *K*_D_ value of 28 μM for CFH self-association (23) and the *K*_D_ value of about 10 μM for CFH–zinc binding and about 100 μM for C3b–zinc binding indicate even weaker affinities (22). The latter interactions are more relevant to drusen formation (retinal deposits of aggregated proteins and oxidized lipids associated with AMD) (37) than to the CFH–heparin interaction at host cell surfaces.

Other groups have reported *K*_D_ values for the CFH–heparin interaction. That of 9.2 nM was reported for CFH binding to unfractionated heparin in 50 mM Na phosphate, 100 mM NaCl buffer, pH 7.2, while that of 9 μM was reported for CFH SCR-19/20 binding to heparin dp4 in 20 mM acetate, 200 mM NaCl buffer, pH 4 (38, 39). The differences from the values in Table 1 are attributable to the less physiological ionic strengths used in these experiments, these buffers being rather different from that of Dulbecco’s phosphate buffer (137 mM NaCl and 11 mM phosphate, pH 7.4).

## Conclusion and Future Considerations

The immune significance of the CFH–HS interactions has been clarified by our molecular solution structures for heparin, HS, and CFH. We provide a first molecular model of the CFH–HS interaction, i.e., a bivalent and co-operative CFH binding mechanism to heparin and HS exists that clarifies how CFH binds to host cell surfaces. At the cell surface, native HS is comprised of sulfated heparin-like S-domains interspaced by desulfated NA-domains of HS. The heparin solution structures possess semi-rigid extended conformations with notably less flexibility than those found in the flexible extended or bent desulfated HS structures. The solution structure of CFH revealed a folded-back 20-SCR domain structure in which the functional N-terminal and C-terminal domains are extended outwards from a compact core of shorter glycosylated SCR domains with longer inter-SCR linkers. Unlike the NA-domains of HS, there is no evidence for significant flexibility in full-length CFH. Consequently, the combination of the non-flexible CFH and heparin dp18–dp36 structures *in vitro* leads to the formation of ring-like models for their complexes (Figure 7). *In vivo*, the views of Figure 7 are readily transformed to suggest how a CFH monomer binds to two different S-domains on a host cell surface (Figure 9B). The flexibility of the NA-domains may facilitate optimal binding of the CFH and S-domains at the cell surface.

Such a bivalent CFH binding mechanism to HS has implications for immune function. In theory, the combination of two separate weak binding events with micromolar affinities becomes a much strengthened interaction if both weakly bound CFH sites bind simultaneously to HS at a cell surface. This prediction was confirmed by surface plasmon resonance studies of full-length CFH and its functional fragments to immobilized heparin on sensor chips. Such a binding interaction may position CFH SCR-1/4 away from the cell surface to facilitate their binding to surface-bound C3b for its regulation. The binding of CFH at SCR-19/20 to the cell surface may orientate CFH into a position that is optimal for its SCR-1/4 domains to bind to C3b (Figure 9B).

For reason of co-operativity, CFH is envisaged to bind preferentially to surfaces showing the right spatial density of anionic oligosaccharides. This implies that the moderately strong binding of CFH to host cell surfaces may differ between different cell types or degrees of sulfation. If HS binding is reduced at either SCR-7 or SCR-20 in CFH, co-operativity implies that there will be a disproportionate effect on CFH regulatory function. In CFH-associated genetic diseases such as AMD and aHUS, the CFH–heparin interaction may be affected by polymorphisms or mutations. Disease-risk polymorphisms will exert their effect over a period of decades, while disease-causing mutations will show a much earlier effect during a lifespan. The AMD-risk CFH polymorphism Y402H occurs in 33% of individuals. AMD occurs primarily in the aged population, this being responsible for over 50% of blindness in the elderly in the Western world (40). Mutations leading to aHUS occur mostly in the C-terminal SCR-19/20 domains of CFH, and aHUS is a common cause of renal failure in young children (41).

The biochemical mechanism of CFH-associated genetic disease may involve either the facilitation of CFH aggregation to form pathogenic deposits, or the biochemical loss of CFH regulatory control (42). Present evidence for either mechanism is not definitive. In terms of an aggregation mechanism, CFH has been found in drusen that are a hall-mark of AMD (43). Glycosaminoglycans have also been identified within Bruch’s membrane, although their size and structure in drusen is not yet known (44). The availability of free S-domains in glycosaminoglycans with size dp18 or more may lead to the formation of ring-like CFH–heparin aggregates, e.g., if these S-domains are released from the cell surface during inflammatory attack. Polyanions also cause CFH to aggregate (45). In terms of an alternative mechanism based on reduced inflammatory regulation that may lead to disease, CFH disease-causing mutations have been summarized on the web (46). These affect each of the two CFH–heparin binding sites:

(i) Three studies of the Tyr402His polymorphism in SCR-7 show that CFH His402 binds more weakly to heparin than CFH Tyr402 (47–49). If so, the weaker binding of CFH His402 to the heparin-like S-domains of HS would compromise both the CFH interaction with C3b and the bivalent binding of CFH to cell surfaces (Figure 9C). This scenario is similar to that proposed for the CFH–CRP interaction (6) in that the weaker binding of CFH His402 to host cell surfaces would predispose toward greater inflammatory damage. However, other studies reported variable outcomes depending on the heparin preparation in use and its degree of sulfation (50, 51), while another study reported that no significant difference was observable between the Tyr402 and His402 allotypes (52). In opposition to these results, the crystal structure of SCR-6/8 His402 bound to a heparin analog suggests that the S-domains bind more strongly to the His402 allotype than to the Tyr402 allotype (53).
(ii) For C-terminal CFH mutations in SCR-19/20 that lead to aHUS, these occur mostly in young individuals, often being triggered by an immune insult to the kidney such as a bacterial infection (41). aHUS is primarily caused by mutations within SCR-19/20, often those affecting heparin binding or C3d-binding properties (46). The C-terminal mutations may affect CFH function by perturbing the orientation of SCR-1/4 relative to C3b when CFH is bound to the host cell surface (Figure 9C).

It is not clear at present why a polymorphism at SCR-7 in CFH leads to one immune disease, while mutations at SCR-19/20 lead to a different disease altogether. Further developments to elucidate the molecular mechanism for CFH binding to native HS may lead to new therapeutic approaches for diseases such as AMD or aHUS.

## Conflict of Interest Statement

The authors declare that the research was conducted in the absence of any commercial or financial relationships that could be construed as a potential conflict of interest.

## Abbreviations

aHUS, atypical hemolytic uremic syndrome; AMD, age-related macular degeneration; CFH, complement factor H; CUB, complement C1r/CIS–UEGF–BMP1 domain of C3; *K*_D_, dissociation constant; SCR, short complement regulator; TED, thioester domain of C3.
